# Supplementary material for: Genetic Predisposition to Elevated Levels of Circulating ADAM17 Is Associated with the Risk of Severe COVID-19
Source: Int J Mol Sci. 2023 Nov 1;24(21):15879. doi: 10.3390/ijms242115879 (PMC10647461; doi:10.3390/ijms242115879)
Supplement: Supplementary file 1 [file ijms-24-15879-s001.zip › ijms-2652995-SI.pdf]

Supplemental Table S1. Genome-wide association summary statistics used in the MR analyses.

Trait	Name of the dataset	Sample size	Consortium or project name	PMID	link
ADAM17 (extracellular)	8959_61_ADAM17	35 559	Icelandic Cancer Project and deCODE	34857953	https://www.decode.com/summarydata/
ADAM17 (cytoplasmic)	8821_1_ADAM17	35 559	Icelandic Cancer Project and deCODE	34857953	https://www.decode.com/summarydata/
Critical ill COVID-19	A2_ALL_eur_leave_23andme	1 086 211	COVID-19 host genetics initiative	34237774	https://www.covid19hg.org/results/r7/
Hospitalized COVID-19	B2_ALL_eur_leave_23andme	2 095 324	COVID-19 host genetics initiative	34237774	https://www.covid19hg.org/results/r7/
SARS-CoV-2 reported infection	C2_ALL_eur_leave_23andme	2 597 856	COVID-19 host genetics initiative	34237774	https://www.covid19hg.org/results/r7/
body mass index (BMI)	bmi.giant-ukbb.meta-analysis.combined	806 834	UKB and GIANT	30239722	https://portals.broadinstitute.org/collaboration/giant/images/1/14/Bmi.giant-ukbb.meta-analysis.combined.23May2018.HapMap2_only.txt.gz

Supplemental Table S2. Variants associated with ADAM17 and associated traits observed in the PhenoScanner database.

Exposure	SNP	Chr	Pos	Pval	Associated Traits
ADAM17	rs10922098	1	196 664 651	9.36×10-6	forced vital capacity
(extracellular)	rs7549171	1	197 177 632	1.23×10-113	age-related macular degeneration
	rs1355538	3	165 505 177	3.50×10-150	serum butyrylcholinesterase activity
	rs6457457	6	31 878 108	3.77×10-06	peak expiratory flow
	rs444921	6	31 932 177	8.70×10-62	rheumatoid arthritis
	rs28688825	6	32 587 157	1.80×10-92	rheumatoid arthritis
ADAM17	rs374896	1	196 692 378	5.72×10-06	forced vital capacity
(cytoplasmic)	rs17209907	6	32 446 261	6.67×10-82<br>3.77×10-58<br>3.80×10-51	white blood cell count<br>sum neutrophil eosinophil counts<br>sum basophil neutrophil counts
	rs12156434	9	124 133 218		-
	rs55701306	17	16 842 447		-

SNP: single nucleotide polymorphism; Chr: chromosome; Pos: position; Pval: p-value was provided from PhenoScanner (http://www.phenoscanner.medschl.cam.ac.uk/)

Supplemental Table S3. Results from MR analysis estimating causal effects for circulating ADAM17 on COVID-19.

Exposure	Outcome	Method	#SNPs	beta	se	pval	mean F statistic	I^2 (unweighted)
ADAM17 (extracellular)	Critical ill COVID-19	MR Egger	6	0.38	0.42	0.419	89.98	0.88
		IVW	6	0.23	0.10	0.025		
		Weighted median	6	0.25	0.10	0.016		
		RAPS	23	0.13	0.07	0.041	39.76	0.84
	Hospitalized COVID-19	MR Egger	6	0.35	0.19	0.146	89.98	0.88
		IVW	6	0.09	0.05	0.090		
		Weighted median	6	0.11	0.06	0.079		
		RAPS	23	0.02	0.04	0.614	40.20	0.84
	SARS-CoV-2 reported infection	MR Egger	6	0.06	0.09	0.531	89.98	0.88
		IVW	6	0.01	0.03	0.584		
		Weighted median	6	0.01	0.03	0.723		
		RAPS	22	-0.01	0.02	0.719	40.76	0.85
ADAM17 (cytoplasmic)	Critical ill COVID-19	MR Egger	3[a]	-0.45	1.27	0.782	25.30	0.75
		IVW	3[a]	-0.08	0.26	0.758		
		Weighted median	3[a]	0.00	0.25	0.993		
		RAPS	17	-0.20	0.11	0.055	23.41	0.69
	Hospitalized COVID-19	MR Egger	3[a]	-0.45	0.67	0.626	25.30	0.75
		IVW	3[a]	-0.17	0.16	0.283		
		Weighted median	3[a]	-0.12	0.16	0.466		
		RAPS	17	-0.05	0.07	0.467	23.33	0.70
	SARS-CoV-2 reported infection	MR Egger	4	-0.02	0.40	0.966	30.36	0.62
		IVW	4	-0.06	0.09	0.524		
		Weighted median	4	-0.01	0.08	0.900		
		RAPS	17	-0.03	0.03	0.304	23.38	0.70

[a] rs17209907 is not present in the outcome GWAS. IVW: Inverse-variance weighted; RAPS: robust adjusted profile score; I^2: an adapted I-squared statistic to assess violation of the "NO Measurement Error" (NOME) assumption for instruments used for MR-Egger.
regression.

Supplemental Table S4. Heterogeneity and directional horizontal pleiotropy.

		Heterogeneity		Horizontal pleiotropy		
Exposure	Outcome	Cochran's Q	P	Egger intercept	SE	P
ADAM17 (extracellular)	Critical ill COVID-19	9.14	0.10	-0.014	0.039	0.74
	Hospitalized COVID-19	4.42	0.49	-0.025	0.018	0.24
	SARS-CoV-2 reported infection	3.18	0.67	-0.005	0.009	0.61
ADAM17 (cytoplasmic)	Critical ill COVID-19	2.97	0.23	0.023	0.075	0.81
	Hospitalized COVID-19	2.72	0.26	0.017	0.039	0.74
	SARS-CoV-2 reported infection	8.15	0.04	-0.002	0.023	0.93

P: p-value; Egger intercept: MR-Egger intercept ; SE: standard error;

Supplemental Table S5. Results from MR analysis estimating causal effects for COVID-19 on circulating ADAM17.

Exposure	Outcome	Method	#SNPs	beta	se	pval	mean F statistic	I^2 (unweighted)
Critical ill COVID-19	ADAM17 (extracellular)	MR Egger	37	0.03	0.02	0.278	67.15	0.93
		IVW	37	-0.02	0.01	0.034		
		Weighted median	37	-0.02	0.02	0.199		
		RAPS	107	-0.01	0.01	0.342	38.06	0.88
Hospitalized COVID-19		MR Egger	40	0.04	0.05	0.461	51.25	0.85
		IVW	40	-0.04	0.02	0.048		
		Weighted median	40	-0.02	0.03	0.362		
		RAPS	141	0.01	0.01	0.572	30.48	0.77
SARS-CoV-2 reported infection		MR Egger	16	-0.07	0.10	0.472	72.00	0.94
		IVW	16	-0.03	0.05	0.486		
		Weighted median	16	-0.05	0.07	0.424		
		RAPS	78	-0.02	0.03	0.553	33.17	0.86
Critical ill COVID-19	ADAM17 (cytoplasmic)	MR Egger	37	0.00	0.02	0.90	67.15	0.93
		IVW	37	-0.01	0.01	0.19		
		Weighted median	37	0.01	0.02	0.76		
		RAPS	107	0.00	0.01	0.63	38.06	0.88
Hospitalized COVID-19		MR Egger	40	0.04	0.05	0.43	51.25	0.85
		IVW	40	-0.01	0.02	0.75		
		Weighted median	40	0.02	0.03	0.38		
		RAPS	141	0.01	0.01	0.32	30.48	0.77
SARS-CoV-2 reported infection		MR Egger	16	0.05	0.10	0.60	72.00	0.94
		IVW	16	0.04	0.05	0.45		
		Weighted median	16	0.04	0.07	0.53		
		RAPS	78	-0.01	0.03	0.73	33.17	0.86

IVW: Inverse-variance weighted; RAPS: robust adjusted profile score; I^2: an adapted I-squared statistic to assess violation of the "NO Measurement Error" (NOME) assumption for instruments used for MR-Egger. regression.

Supplemental Table S6. Summarizing suggested circulating proteins being causally associated with an increased risk of critical ill COVID-19.

Protein (exposure)	Protein assay	OR	Lower 95% CI	Upper 95% CI	Average F-statistic
GCNT4_Sun	SOMAscan	1.35	1.26	1.44	50.8
CD207_Sun		1.17	1.11	1.23	95.1
RAB14_Sun		1.22	1.16	1.28	95.0
C1GALT1C1_Sun		1.21	1.13	1.28	42.3
ABO_Sun		1.12	1.08	1.16	443.9
ADAM17_extracelluar_domain	SOMAscan	1.26	1.03	1.55	90

OR: odds ratio; CI: confidence interval
